# Route bundling in polygonal domains using Differential Evolution

**DOI:** 10.1186/s40638-017-0079-x

**Published:** 2017-12-08

**Authors:** Victor Parque, Satoshi Miura, Tomoyuki Miyashita

**Affiliations:** 1 Department of Modern Mechanical Engineering, Waseda University, 3-4-1 Okubo, Shinjuku-ku, Tokyo, 169-8555 Japan; 2 Department of Mechatronics and Robotics, Egypt-Japan University of Science and Technology, Qesm Borg Al Arab, Alexandria, 21934 Egypt

**Keywords:** Route bundling, Differential Evolution, Path planning

## Abstract

Route bundling implies compounding multiple routes in a way that anchoring points at intermediate locations minimize a global distance metric to obtain a tree-like structure where the roots of the tree (anchoring points) serve as coordinating locus for the joint transport of information, goods and people. Route bundling is a relevant conceptual construct in a number of path-planning scenarios where the resources and means of transport are scarce/expensive, or where the environments are inherently hard to navigate due to limited space. In this paper we propose a method for searching optimal route bundles based on a self-adaptive class of Differential Evolution using a convex representation. Rigorous computational experiments in scenarios with and without convex obstacles show the feasibility and efficiency of our approach.

## Background

In this paper we tackle the route bundling problem which consists of compounding multiple routes in a way that intermediate points minimize a global distance metric of multiple origin–destinations pairs. In this context, the ultimate goal in route bundling is to construct tree-like graph structures where the anchoring points, being roots of the tree structure, serve as coordinating locus for the joint transport of information, goods and people.

**Fig. 1:**
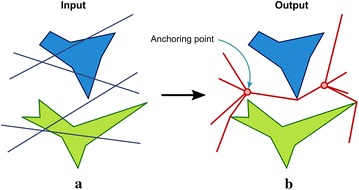
Basic idea of route bundling. Given a polygonal map and edges representing desirable origin–destination pairs, the goal is to find optimal anchoring points minimizing the global distance metric of the bundled route. **a** Bipartite graph and obstacles, **b** route bundling

A fundamental problem behind the construction of route bundles lies in deciding the locations of roots and intermediate nodes to form the optimal tree structures. Also, the presence of obstacles makes the problem non-trivial due to the non-convexity of the search space, thus being hard to deal with analytical and statistical methods. In order to exemplify the conceptual framework involved in route bundling, Fig. 1a shows a bipartite graph which denotes transport needs between origin–destination pairs (which is normally known a priori). Here, nodes of the bipartite graph denote locations for origin and destination, while edges denote needs for transport/communication. Figure 1b shows the bundled route which represents the tree structure aiming at minimizing the global distance metric while avoiding obstacle collision. Here, note that anchoring points are located at some intermediate region of the origin–destination pairs.

### Application background

Compared to the path-planning problem with single origin–destination nodes, the route bundling problem is a generalized formulation in the sense that the latter considers multiple origin–destination pairs. Naturally, in path planning with single origin–destination pairs, the anchoring point (root of minimal tree) coincides with the origin–destination nodes. In the literature, the path planning is a well-studied topic [1–6]; yet, the route bundling problem is an emergent research topic having potential applications in problem scenarios involving compounded path planning with multiple origin–destination pairs. Here, designing the optimal network is relevant for the efficient use of resources while integrating and coordinating transport/communication needs.

Concretely speaking, route bundling has specific applications in environments where resources or means of transport/communication are scarce or expensive. For instance, consider the design of a network for transporting goods from/to multiple areas in an environment covered with obstacles; naturally, since one-to-one transport would cause unwanted traffic or excessive cost in network construction, one is interested in designing a network where intermediate nodes serve as coordinating locus for source/destination locations.

Also, consider the design of optimal wire harness topologies for machines (e.g., cars and ships). Here, free space for electrical wiring is scarce and one-to-one links are rather undesirable; thus, wire harness becomes essential to build tree-like structures aiming to minimize global connectivity while ensuring minimal use of space.

Furthermore, consider the deployment of sensor networks for many-to-many robots in the presence of attenuating obstacles (e.g., disaster areas). Here, attenuating obstacles induce in data loss or deterioration of the ability to communicate (e.g., concrete floors, steel reinforced floors, ceilings, elevators, walls, rock, and reinforced materials). Thus, it becomes imperative to design networks allowing to get the sensor signal around the obstructing materials. In disaster areas, route bundling becomes the building block to enable energy-efficient networks.

Generally speaking, the presence of obstacles and holes in the environment induces on limited space and navigability, thus making route bundling relevant when either transport and communication means are scarce and expensive, or when optimal networking is a goal in many-to-many origin–destination settings.

### Related works

Basically, the algorithmic foundations of the route bundling have been laid out in two different fields: wireless sensor networks and network visualization.

In one hand, the study of wireless sensor networks [7] has rendered methodologies for network protocol and topology construction. Examples include the construction of a connected network to ensure complete coverage of an area of interest with the minimum number of nodes as possible [8], the degree-constrained minimum-weight connected dominated set for energy-efficient topology control in wireless sensor networks [9], the topological optimization for consensus-based clock synchronization protocols [10], the tree topology construction for heterogeneous wireless sensor networks [11], the self-stabilizing algorithms to construct rooted trees under the assumption of node disconnection [12] and a number of topology optimization for network coverage, connectivity, energy savings, delay minimization, optimal routing and broadcasting [13–17].

In other domains, the route bundling problem has its closest foundations in edge bundling for network visualization field. Here, the basic aim is to compound edges in complex networks to ease the visualization or rendering of large-scale networks. In particular, the conventional works have focused on the geometry-based edge clustering, in which the edges in the graph are forced to pass through points in a control mesh [18]. Also, the force-based edge bundling where edges are modeled as springs being able to attract to each other [19, 20]. Furthermore, hierarchical clustering approaches have emerged. Here, in [21], the authors describe an approach based on attraction to the skeleton of the adjacent edges. And, in [22], the authors describe a kd-tree-based optimization of the centroid points of close edges in the graph.

### Our contribution

Although the above described algorithms for topology optimization in wireless sensor networks aim at finding an optimal hierarchy given a number of nodes, the existing algorithms are irrelevant to our scope since route bundling does not require centralized communication and compliance with network coverage (e.g., hoping diameter).

On the other hand, existing algorithms for edge bundling have a different scope: network bundles aim at rendering aesthetically pleasing and topologically compact drawings; yet, the existing algorithms do not necessarily aim at minimizing a global distance metric. Furthermore, it is non-trivial for the existing edge bundling algorithms to obtain minimal-length networks in the presence of obstacles and holes in the environment.

Thus, in order to fill the above gaps, and having a different scope, we focus on the problem of designing optimal route bundles. In order to tackle this problem, we use Differential Evolution embedded with a convex representation of the search space (free region) to optimize route planning and bundling in polygonal domains. The basic idea of our approach is to search over the space of a convex representation of a polygonal map by sampling with self-adaptive interpolation vectors. Here, the unique point of our approach is to balance the explorative and exploitative sampling of anchoring points while explicitly avoiding the computations of point inside polygons. Our contributions are summarized as follows:

- We propose a nature-inspired algorithm for searching tree bundles aiming at minimizing global length in a polygonal domain. In our approach, we use Differential Evolution and study the performance of our proposed algorithmic variants including the following:
  - DENC, Differential Evolution with Neighborhood and Convex Representation.
  - DEN, Differential Evolution with Neighborhood.
  - DEC, Differential Evolution with Convex Representation.
  - DE, Differential Evolution without Neighborhood nor Convex Representation.
- We perform more than 12,000 experimental evaluations to confirm the feasibility, efficiency and robustness of our approach by considering diverse number of edges in the input bipartite network, diverse complexity configurations of polygonal obstacles with convex and non-convex geometry (number of edges in polygon up to 10), and parametric comparisons considering population size and neighborhood size. Furthermore, we compare the convergence performance of the above described algorithmic variants. Based on these computational experiments, we provide insights on how to design optimal tree bundles by using our nature-inspired approach.

In the rest of the paper, we describe our framework in “Methods” section, and then we describe our results and provide insights from our computational experiments in “Computational experiments” section, and finally conclude our paper in “Conclusions” section.

**Fig. 2:**
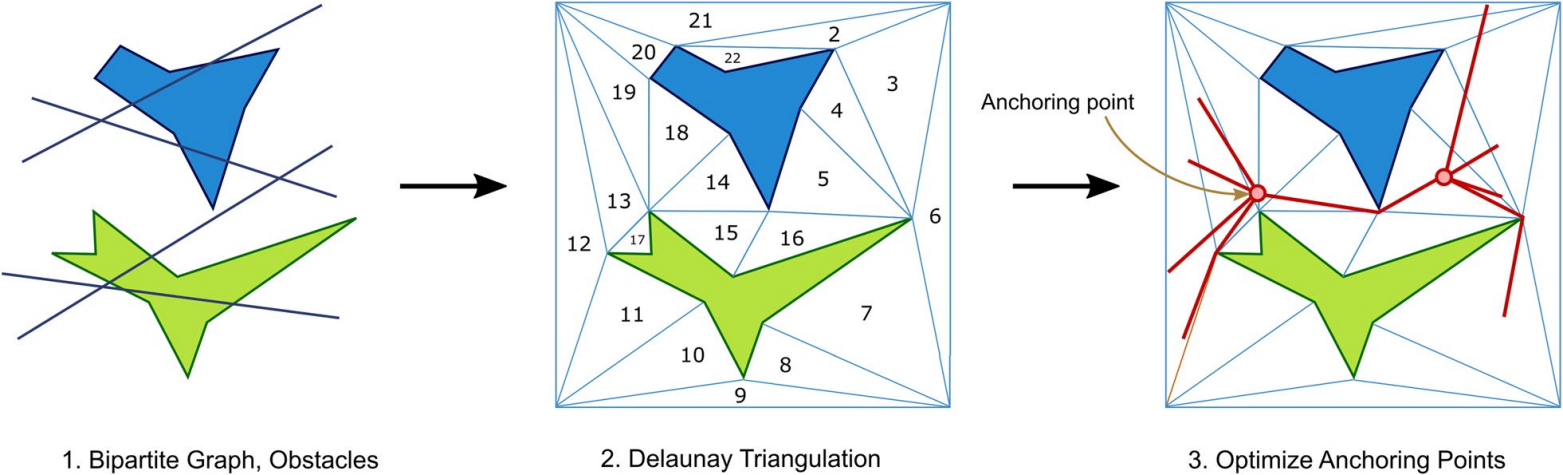
Basic steps in the proposed approach. Inputs consist of a polygonal map and a bipartite network, wherein edges of the bipartite network represent desirable source–destination pairs. The output is a tree-like network with anchoring points at intermediate points optimizing a global distance metric

## Methods

This section describes the basic ideas as well as the algorithmic foundations in our proposed approach for route bundling.

### Basic framework

The basic concept of our proposed approach is depicted by the following equation:

1 $$\begin{aligned} &\underset{x}{{\mathbf{Minimize }}}&F(x) \\&\text {{subject to}}&x \in {{\mathbf {T}}}\end{aligned}$$

where *x* is the *encoding* (representation) of the route bundle, *F*(*x*) is the distance metric which is used to evaluate the quality/fitness of the bundled routes, and ${\mathbf {T}}$ is the search space of *feasible* route bundles.

In the above definition, the *encoding* implicitly represents a tree structure whose edges are free of overlaps with obstacles.

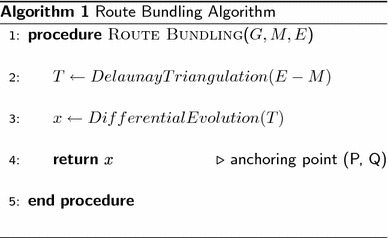



Also, note that the constraint $x \in {\mathbf {T}}$ makes explicit the requirement that optimization is realized within the space ${\mathbf {T}}$ of *feasible* route bundles. In latter sections, we describe the representation which allows sampling of *feasible* route bundles.

Then, in order to solve the above problem, a set of explicit a priori knowledge is considered fundamental. In our study, we assume to have knowledge of the following elements (as shown by Fig. 2):

- Definition of a bipartite graph $G = (V,E)$ wherein the edge $e \in E$ represents the origin–destination pair (implying needs for communication/transportation between two points). Here, the number of edges and the locations of the source–destination pairs in the graph *G* are defined by the characteristics of the environment and/or by the needs of the user or network designer.
- Definition of locations and geometry of the obstacles in the environment (which denote unfeasible areas for navigation/transportation). For simplicity and without loss of generality, we use polygonal obstacles with/without convexity properties which are reminiscent of indoor environments.

In order to give a glimpse of the algorithmic flow in our proposed approach, Fig. 2 and Algorithm 1 show the basic steps for route bundling. Basically, our approach consists as follows:

- First, the geometry of the free space is computed given information of the set of obstacles (map) and bipartite network.
- Then, the free space is triangulated using the Delaunay approach.
- Finally, the locations of the anchoring points are optimized within the triangulated free space. Here, during optimization, fitness is defined by the distance metric *F*(*x*).

In the following, we describe the fundamental concepts involved in our approach: (1) the *encoding* (representation) of bundled routes, (2) the distance metric, as well as (3) the optimization method to solve Eq. (1).

### Representation of bundled routes

This subsection describes the mechanism used to represent bundled routes.

Given a bipartite graph $G = (V, E)$ with edges representing origin–destination pairs, the reader may note that a bundled route can be easily represented by the coordinates of a pair of anchoring points connecting all origins and destinations, as Fig. 1 shows. By using this concept, whenever the coordinates of the vertices $v \in V$ of the bipartite graph *G* are presented in $\mathbb {R}^2$, then the route bundling can be easily represented by the following 4-element tuple:

2 $$\begin{aligned} x = (P_x, P_y, Q_x, Q_y) \end{aligned}$$

where $P_x$ and $P_y$ are the coordinates in the x-axis and y-axis, respectively, in which the anchoring point nearest to the origins is located at $(P_x, P_y)$, and the anchoring point nearest to the destination is located at $(Q_x, Q_y)$. The reader may easily note that $x \in \mathbb {R}^4$ holds.

The above representation is simple; yet, it has a fundamental problem: it is unable to encode *feasible* route bundles since the condition $P,Q \in \mathbb {R}^2$, implying $P = (P_x, P_y)$ and $Q = (Q_x, Q_y)$, does not ensure that coordinates are outside the non-navigable space (overlapping with obstacles).

In order to tackle the above problem, we propose representing the coordinates of the anchoring points by using only the navigable free space. The basic concept is as follows.

- The free space of the polygonal map is triangulated using the Delaunay approach [23], as Fig. 2 exemplifies, in which a set $T = \{t_1, t_2, \ldots , t_i, \ldots , t_n\}$ of *n* triangles are obtained.
- Then, the anchoring points can be represented by 3-element tuples, as follows:
  3 $$\begin{aligned} P = {(i, r_1, r_2) } \end{aligned}$$
  where $i \in [n]$ and $r_1, r_2 \in [0,1]$. In the above *encoding*, *i* is the index of the *i*-th triangle $t_i \in T$, and $r_1, r_2$ are real numbers in the interval [0, 1].

The unique feature of the above *encoding* lies in the ability to represent arbitrary points in $\mathbb {R}^2$ which guarantee to be inside the (free) navigable space. Note that the following relation holds:

$$\begin{aligned} P \in {\mathbb {N}^{[n]}} \times \mathbb {R}^{[0,1]} \times \mathbb {R}^{[0,1]} \end{aligned}$$

Furthermore, for a polygonal map in 2-D, the equivalent cartesian coordinates can be computed by using the following relation [24]:

4 $$\begin{aligned} (P_x, P_y) = (1-r_1)A_i + \sqrt{r_1}(1-r_2)B_i + \sqrt{r_1}r_2C_i \end{aligned}$$

where $A_i, B_i, C_i$ are the 2-dimensional coordinates of the vertices of the *i*-th triangle $t_i \in T$. Intuitively, $r_1$ represents the percentage from vertex $A_i$ to the opposing edge in the triangle $t_i \in T$. The square root of $r_1$ has the role of considering a uniform random point with respect to the triangle area. Although it is possible to use the simple barycentric interpolation, the above representation has the added benefit of enabling the uniform sampling of arbitrary points.

Then, for a route bundle with two connected anchoring points *P* and *Q*, in which *P* connects to the origin nodes, and *Q* connects to the destination nodes (see Fig. 2 for a basic reference), it is possible to use Eq. 3, to deduce an encoding for route bundles by using a 6-element tuple, as follows:

5 $$\begin{aligned} x = (i^P, r^P_1, r^P_2, i^Q, r^Q_1, r^Q_2) \end{aligned}$$

where $i^P, i^Q$ are natural numbers in the interval [*n*], and $r^P_1, r^P_2, r^Q_1, r^Q_2$ are real numbers in the interval [0, 1]. Basically, the above expression has the role of representing two coordinates in the plane, and for simplicity, we denote the search space $x \in {\mathbf {T}}$, wherein the following holds:

$$\begin{aligned} {\mathbf {T}}\,\equiv & {}\, {\mathbb {N}^{[n]}} \times \mathbb {R}^{[0,1]} \times \mathbb {R}^{[0,1]} \times {\mathbb {N}^{[n]}} \times \mathbb {R}^{[0,1]} \times \mathbb {R}^{[0,1]} \end{aligned}$$

Thus, by using a triangulation of the free space and the relations of Eqs. (3)–(5), it becomes possible to sample arbitrary points uniformly in the *convex* search space ${\mathbf {T}}$. Intuitively, the above representation allows to render *feasible* route bundles efficiently since,

- Bijection to Cartesian coordinates is possible in *O*(1) by using Eq. 4.
- Route bundles are guaranteed to avoid overlaps with obstacles, and
- Explicit computation of point inside polygon is avoided, implying the efficiency in scalability while sampling a very large number of points in the free navigable space.

### Cost function

In this subsection, we describe the distance metric used to measure the quality/fitness of route bundles.

Once the search space $x \in {\mathbf {T}}$ is constructed, our next goal is to find anchoring points *P* and *Q* (as shown by Fig. 2) which minimize a distance metric. For simplicity and without loss of generality, we use the following metric:

6 $$\begin{aligned} F(x) = \sum _{e \in E}d(e_o,P) + d(P,Q) + \sum _{e \in E}d(Q,e_d) \end{aligned}$$

where *d*(*a*, *b*) is the Euclidean obstacle-free shortest distance metric between points *a* and *b*, $e_o$ is the coordinate of the *origin* node of the edge $e \in E$, $e_d$ is the coordinate of the *destination* node of the edge $e \in E$, and *P* and *Q* are anchoring points being closer to the *origin* $e_o$ and *destination* $e_d$, respectively.

Intuitively, the above cost function represents the euclidean distance between three different groups:

- the distance of the shortest paths between origin nodes to anchoring point *P*,
- the distance of the shortest paths between the anchoring points *P* and *Q*, and
- the distance of the shortest paths between the anchoring points and the destination nodes.

The shortest paths are computed by using A* search [3] and the visibility trace, which is pre-computed from the Delaunay triangulation.

Note that the 2-dimensional coordinates of the anchoring points *P* and *Q* can be computed by combining Eqs. (3)–(5), as follows:

7 $$\begin{aligned} (P_x, P_y) = (1-r^P_1)A^P_i + \sqrt{r^P_1}(1-r^P_2)B^P_i + \sqrt{r^P_1}r^P_2C^P_i \end{aligned}$$

8 $$\begin{aligned} (Q_x, Q_y) = (1-r^Q_1)A^Q_i + \sqrt{r^Q_1}(1-r^Q_2)B^Q_i + \sqrt{r^Q_1}r^Q_2C^Q_i \end{aligned}$$

where $A^P_i, B^P_i, C^P_i$ are the 2-dimensional coordinates of the vertices of the *i*-th triangle $t_i \in T$ where point *P* lies in.

Further extensions are possible. For example, instead of using the above euclidean metric, it is possible to use Manhattan distance (useful for designing networks for integrated circuits and tubular networks inside buildings). Also, it is possible to include weights in the above metric to balance the relevance of the distance to the origins compared to the distance to destinations.

### Differential Evolution

This subsection describes the optimization algorithm used to compute the optimal route bundles.

We use *Differential Evolution* [25, 26] considering global and local interpolation vectors in order to tackle the problem of dealing with multimodal search space (the reader may note that Eq. 6 is multimodal in the case of polygonal maps with non-convex obstacles).

Concretely speaking, the method for sampling in the above mechanism is described by the following equations:

9 $$\begin{aligned} x_{t+1} = {\left\{ \begin{array}{ll} u_t &\quad{} { F(u_t) \le F(x_t)}\\ x_t &{}\quad \text {otherwise} \end{array}\right. } \end{aligned}$$

where

- $x_t$ represents Eq. 5 at iteration *t*, in other words $x_t$ is the *individual* (route bundle),
- *F*(.) is the objective function at Eq. (6) (*minimization*), and
- $u_t$ is the *trial* route bundle solution at iteration *t*.

In the above definition, sampling of new points is realized when the trial vector $u_t$ minimizes or achieves equal performance compared to the current state.

The trial vector $u_t$ is computed from the interpolation of two vectors, as follows:

10 $$\begin{aligned} u_t=\,& {} x^c_t + m_t \circ (v_t - x^c_t ) \end{aligned}$$

11 $$\begin{aligned} m_t=\,& {} [m_{t,1}, m_{t,2}, m_{t,3}, \ldots , m_{t,D}] \end{aligned}$$

- $\circ$ is the *Hadamard* product (element-wise).
- $x^c$ is the *crossover* individual at iteration *t*.
- $v_t$ is the *mutant* individual at iteration *t*.
- $m_t$ is a vector of masks containing zeros and ones.

The mask $m_t$ is computed as follows:

12 $$\begin{aligned} m_{t, j}= & {} {\left\{ \begin{array}{ll} 1, &{}{\mathbf {r}}_{t,j} \le CR \text { or } j = jrand\\ 0, &{}\text {otherwise} \end{array}\right. } \end{aligned}$$

13 $$\begin{aligned} v_t= & {} w^{x_t}.g_t + (1-w^{x_t}).l_t \end{aligned}$$

where

- $\circ$ is the *Hadamard* product (element-wise).
- ${\mathbf {r}}_{t,j}$ and *jrand* are random numbers uniformly distributed in $\mathbb {R}^{[0,1]}$ and $\mathbb {N}^{[D]}$ respectively.
- *CR* is the probability of crossover.
- $D = 6$ is the dimensionality of the route bundling problem, Eq. (5).

In the above definitions, the user may note that high values of the crossover rate *CR* incite higher number of ones in the mask vector $m_t$, thus a highly explorative behavior of the search space.

The *global* and *local* interpolation vectors are computed as follows:

14 $$\begin{aligned} g_t=\, & {} x_t + \alpha ({x_{gbest}} - x_t) + \beta (x^{1} - x^{2}) \end{aligned}$$

15 $$\begin{aligned} l_t= \,& {} x_t + \alpha ({x_{nbest_x}} - x_t) + \beta (x^{p} - x^{q}) \end{aligned}$$

16 $$\begin{aligned} w^{x_t}= \,& {} w^{x_t} + \alpha (w_{gbest} - w^{x_t}) + \beta (w^{1} - w^{2}) \end{aligned}$$

**Fig. 3:**
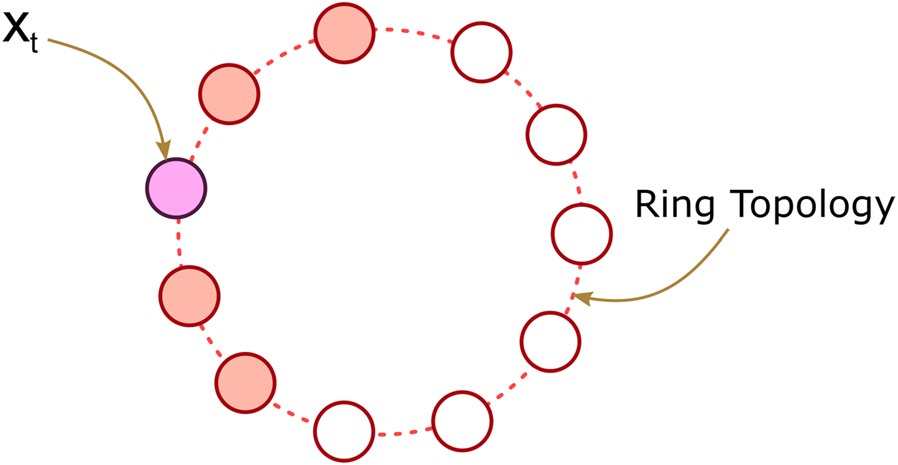
Neighborhood of $x_t$ for $\rho =2$ in a *ring* topology. Spheres denote individuals

where

- $g_t$ is the *global* donor individual.
- $l_t$ is the *local* donor individual.
- $\{x^1_t, x^2_t\} \subset {\mathbf P}$, are random individuals sampled from ${\mathbf P}$ for $x^1 \ne x^2 \ne x_t$.
- $x_{gbest}$ is the *global best* in the population at iteration *t*.
- $x_{nbest_x}$ is the *local best* in the *neighborhood* ${\mathbf N} (x_t)$ of individual $x_t$ at iteration *t*.
- the *neighborhood* ${\mathbf N} (x_t)$ of vector $x_t$ is the set of individuals contiguous to $x_t$ by radius $\rho = \frac{|{\mathbf P} |.\eta }{2}$ in a *ring topology* (see Fig. 3 for a *ring topology* with radius $\rho = 2$).
- $\{x^p, x^q\} \subset {\mathbf N} (x_t)$, are random individuals for $x^p \ne x^q \ne x_t$.
- $w^{x_t}$ denotes the coefficient of individual $x_t$, in which any coefficient $w^{x_t} \in U[0,1]$ is set randomly at initial iteration.
- $w_{gbest}$ is the coefficient associated to $x_{gbest}$,
- $w^{1}, w^{2}$ are the coefficients associated to the vectors $x^{1}, x^{2}$ respectively.

Note that in the above definitions, Differential Evolution uses global and local interpolation vectors $g_t$ and $l_t$, respectively; in which the mutant vector $v_t$ is a linear interpolation between $g_t$ and $l_t$.

The global (local) interpolation vector $g_t$ ($l_t$) represents the position to which the direction of the trial vector should aim at in the 6-dimensional search space, considering information from the population (neighborhood). Thus, the global (local) interpolation vector is the result of the current solution being translated by a sum of two directional vectors, one vector representing the direction to the best solution in the population (neighborhood) and another vector representing an arbitrary direction computed from the difference of two solutions within the population (neighborhood).

The first perturbation vector (the one multiplying $\alpha$) is an *arithmetical recombination operator*, while the second perturbation vector (the one multiplying $\beta$) is a *differential mutation*. The parameters $\alpha$ and $\beta$ have a scaling role toward best and arbitrary directions, respectively. A priori knowledge of problem convexity, uni-modality or multi-modality eases the selection of good values of $\alpha$ and $\beta$. Unimodal and convex (multimodal and non-convex) fitness landscapes would favor values of $\alpha$ ($\beta$) being larger than $\beta$ ($\alpha$) to induce in an exploitative (explorative) behavior in both the global and local neighborhood, and to ease the faster convergence. Without a-prior knowledge of the fitness landscape, it is recommendable to use $\alpha , \beta \in (0, 2)$ to avoid overshooting while sampling in the search space [25, 26]. Furthermore, note that when $\alpha = \beta$ and $w = 1$, the above Differential Evolution is equivalent to the conventional DE/target-to-best/1 strategy [26]; thus, the above algorithm is a generalization in which it considers not only the global population, but also the local neighborhood.

In computing the local neighborhood we use the ring topology to ensure speciation of individuals while preserving efficiency in the computation of best individuals in the neighborhood. An alternative approach is to use a clustering approach in which the neighborhood is defined as a local cluster. Yet, compared to the clustering approach, the ring topology is more efficient since computing the best individuals in the neighborhood takes $O({\mathbf P} )$, while the clustering approach takes $O({\mathbf P} ^2)$ for ${\mathbf P}$ being the population size.

Finally, the use of *Differential Evolution* with *global* and *local* interpolation vectors is advantageous to balance both exploration and exploitation over the entire search space $x \in {\mathbf {T}}$, wherein the trade-off between the global and the local search is self-adapted throughout the iterations.

## Computational experiments

This section discusses our experimental results as well as obtained insights after evaluating the performance of our proposed method by using exhaustive computational experiments in diverse polygonal maps with both convex and non-convex topology.

### Settings

Our computing environment was Intel i7-4930K @ 3.4GHz, MATLAB 2016a.

Table 1 shows the key parameters of Differential Evolution such as the probability of crossover *CR* and the scaling factors $\alpha$ and $\beta$.

The reason of using a crossover probability $CR = 0.5$ is to give equal importance to the search directions obtained from historical search, and those obtained considering local and global interpolations.

Also, without a priori knowledge of problem convexity, uni-modality or multi-modality of the route bundling problem, we choose conservative values of $\alpha$ and $\beta$ to induce smooth balance of exploitation and exploration in both the global and local neighborhood; thus, the scaling factor, $\displaystyle \alpha = \beta = \Big |\frac{ln(U(0,1))}{2} \Big |$, allows to search in small steps when computing the global, the local and the self-adaptive directions.

**Table 1:** Parameters in Differential Evolution

Parameter	Symbol	Value
Probability of Crossover	*CR*	0.5
Scaling factor	$\displaystyle \alpha , \beta$	$\Big |\frac{ln(U(0,1))}{2} \Big |$

### Experimental scenarios

In order to enable a meaningful evaluation of our proposed approach, we consider the following environmental scenarios:

**Table 2:** Experimental scenarios

Variables	Symbol	Values
Edges of bipartite graph	|*E*|	$\{5, 10, 15, 20, 25\}$
Polygonal obstacles		$\{1, 2, 3, 4, 5\}$
Sides in obstacles	*S*	$\{5, 10\}$
Population size	$|{\mathbf P} |$	$\{25, 50, 100, 200\}$
Neighborhood scaling factor	$\displaystyle \eta$	$\{0.1, 0.2, 0.4, 0.8\}$

**Fig. 4:**
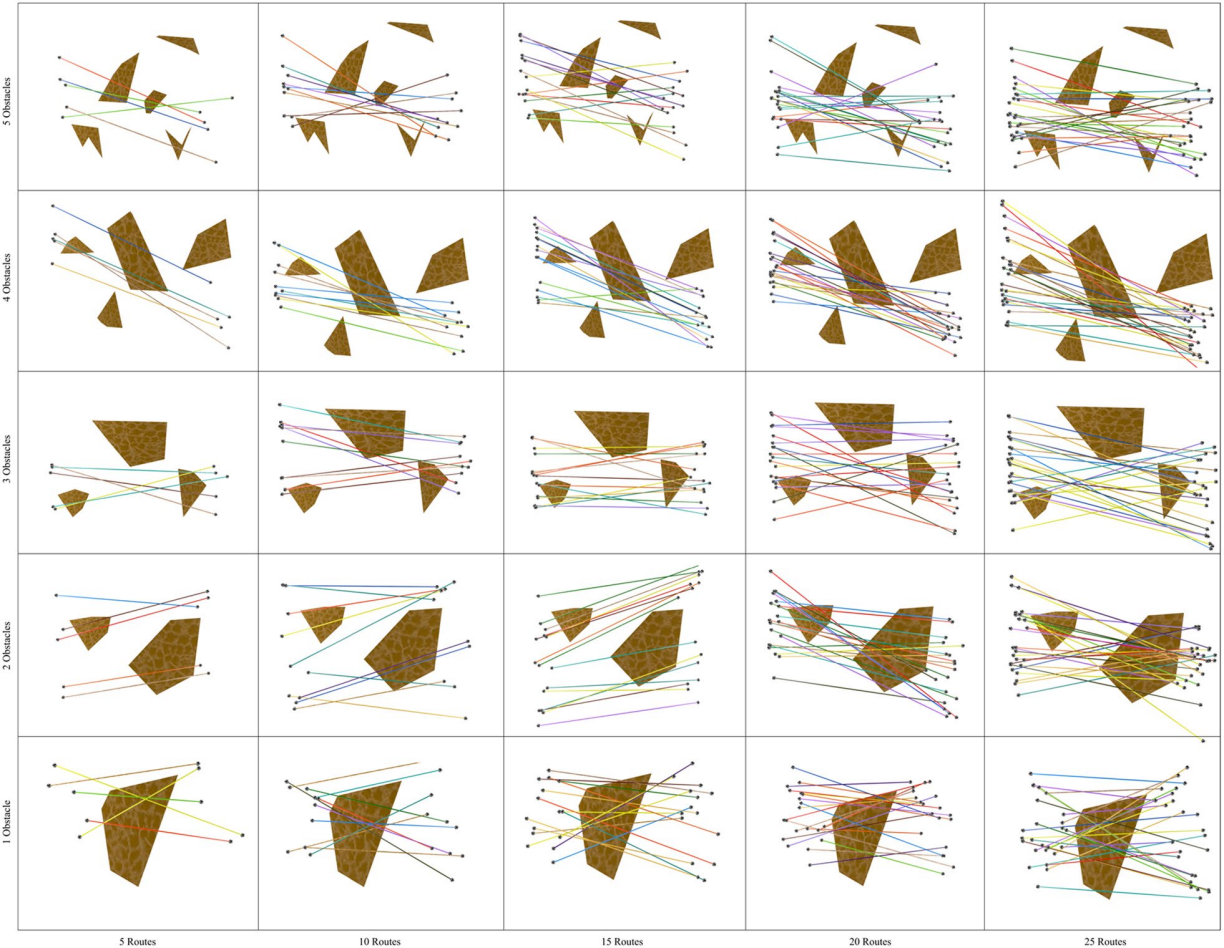
Bipartite network and polygonal map with **5 sides**

**Fig. 5:**
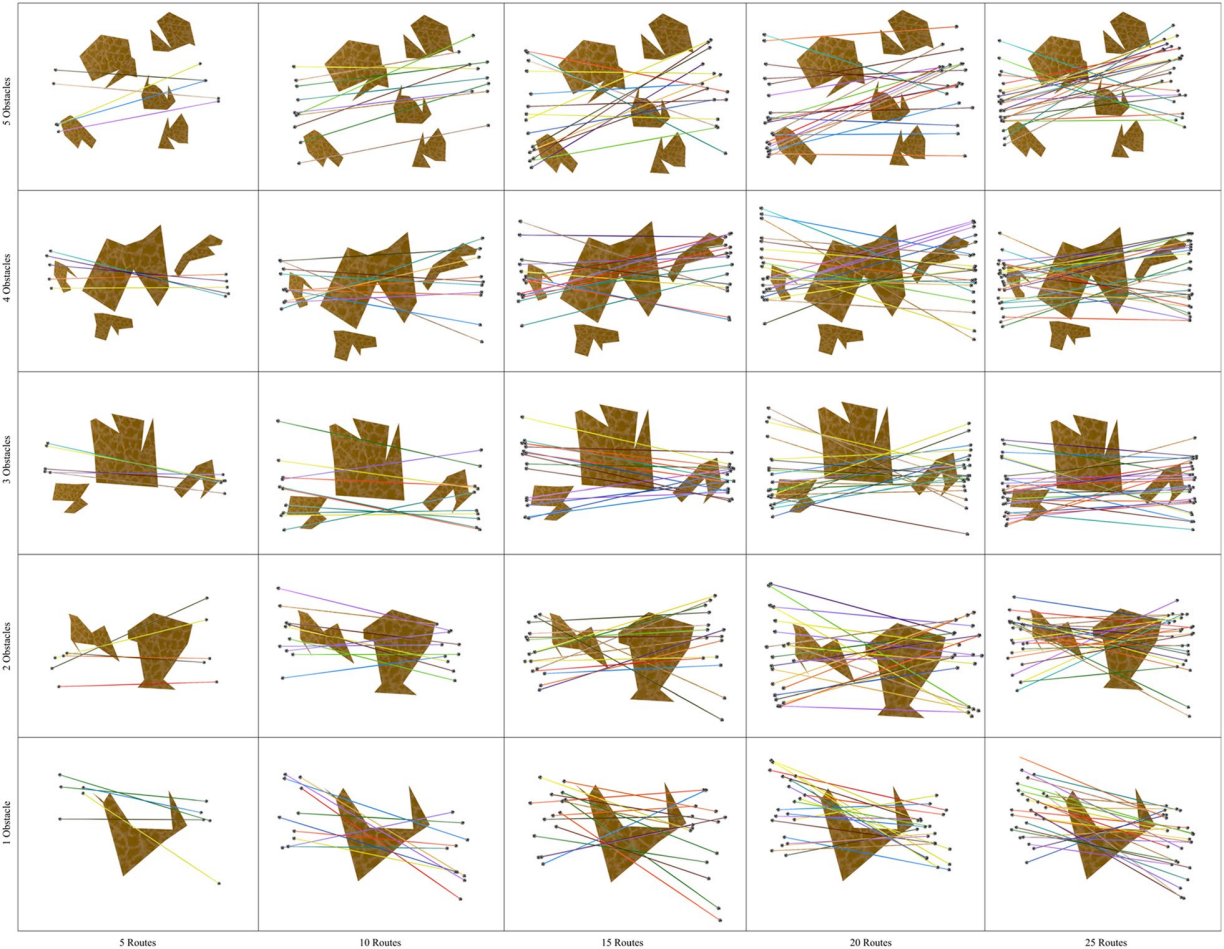
Bipartite network and polygonal map with **10 sides**

In order to give a glimpse of the type of polygonal maps used in our study, Figs. 4 and 5 show the topology of the bipartite network and the polygonal maps. Note that these domains are organized in a grid in which the horizontal (vertical) axis portrays, in ascending order from left (bottom) to right (top), the number of routes (polygons) involved in route bundling.

The main reason of using values of the number of edges |*E*| up to 25 is due to our interest in evaluating the performance in scenarios reminiscent to indoor environments, where the complexity of the environment is controlled by the following elements:

- the number of obstacles in the polygonal map, and/or
- the number of sides for each obstacle.

Thus, complex polygonal domains induce in large number of triangles, edges and vertices in the visibility graph, thus representing a challenging search space for any path-planning algorithm. Our future work aims at using configurations considering large scenarios and being close to outdoor environments.

Furthermore, we considered the following:

- For each combination of the above, 15 independent experiments were performed to solve Eq. 1 by using the optimization algorithm in Eq. 9, and
- For each independent experiment, the maximum number of functions evaluations is set as $10^4$ with initial solutions of route bundles $x_o \in {\mathbf {T}}$ being initialized randomly and independently.

The above is due to our interest in avoiding random bias/luck, and evaluating the efficiency of the proposed method under restrictive computational budget.

As a result of the above considerations, 12,000 experimental conditions were evaluated,^1^ and 11,250,000,000 function evaluations were performed.^2^

**Fig. 6:**
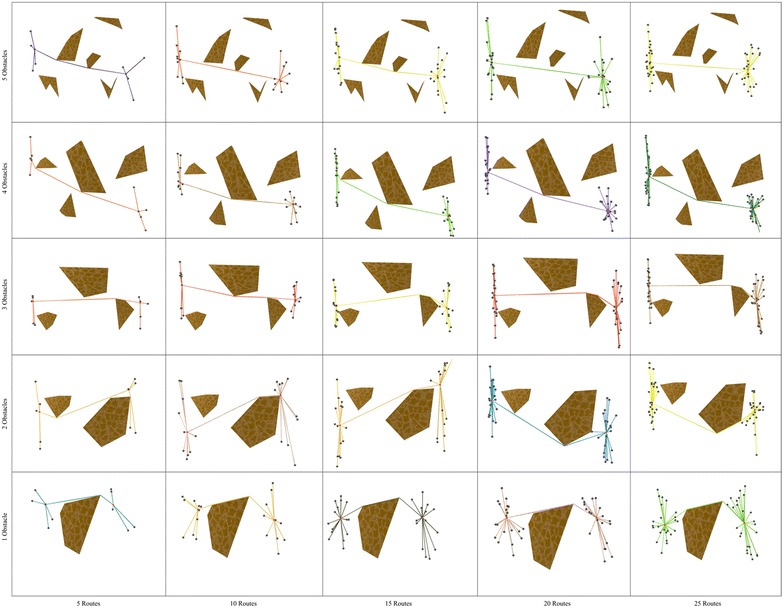
Route bundles in polygonal domains of **S = 5 sides**

**Fig. 7:**
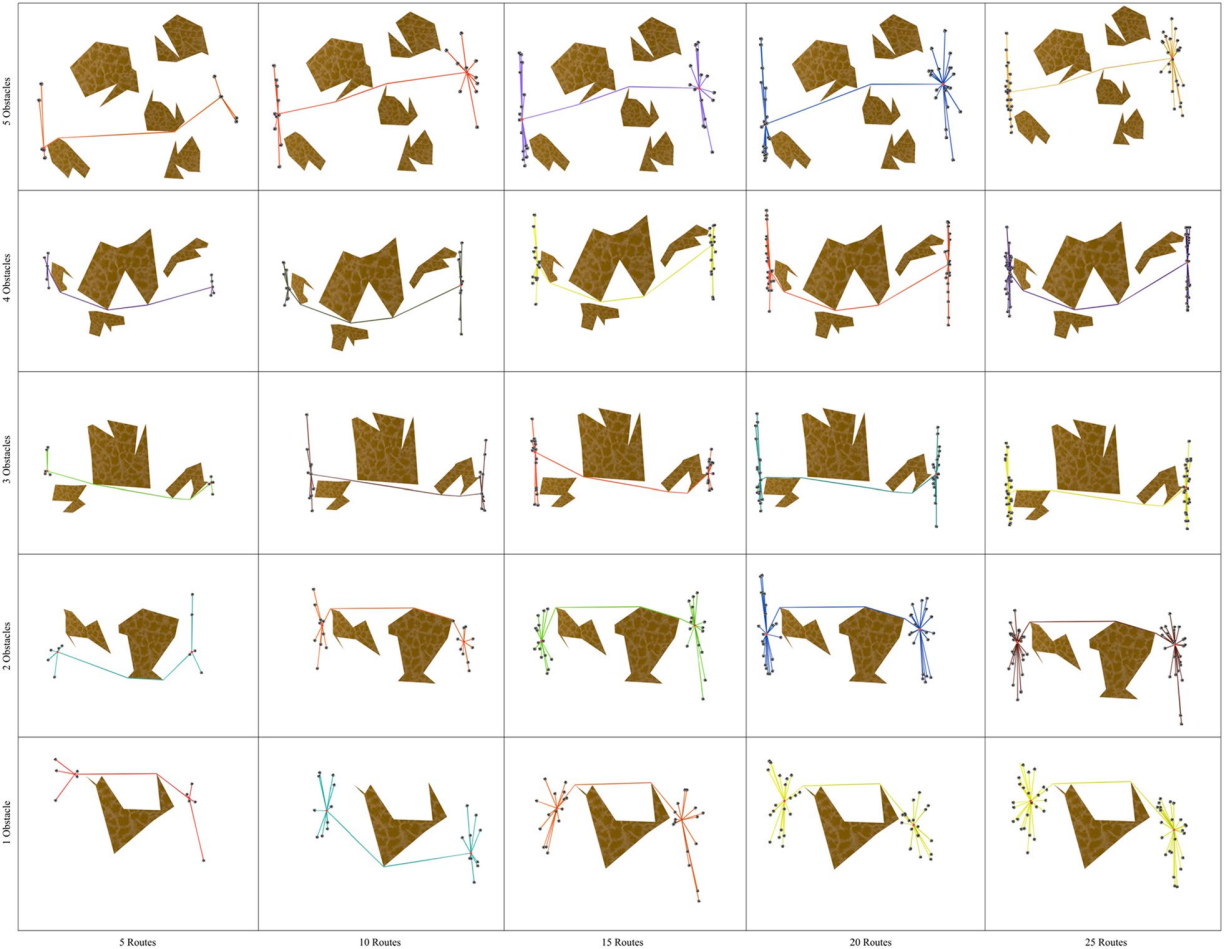
Route bundles in polygonal domains of **S = 10 sides**

**Fig. 8:**
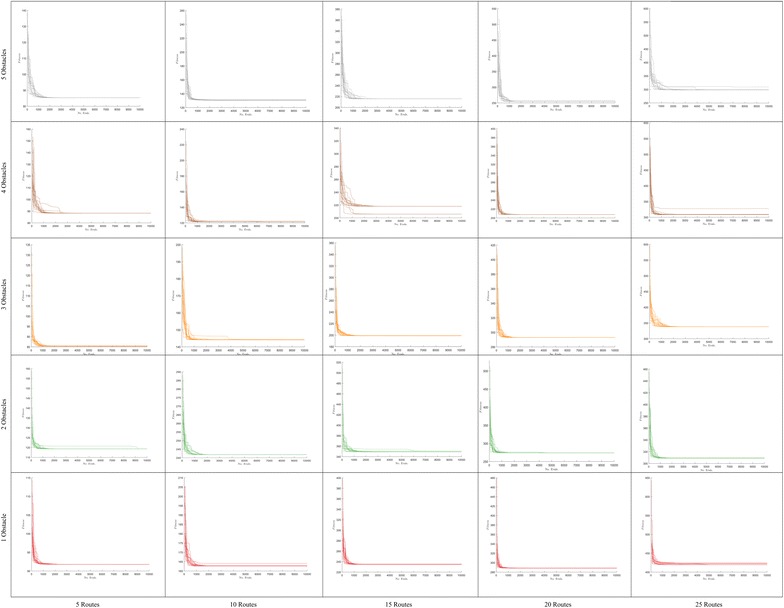
Convergence in polygonal domains of **S = 5 sides**

**Fig. 9:**
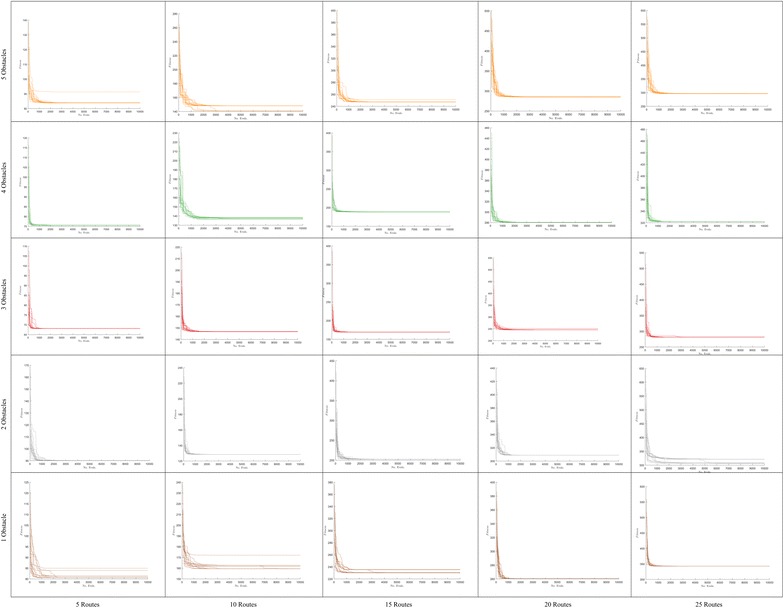
Convergence in polygonal domains of **S = 10 sides**

**Fig. 10:**
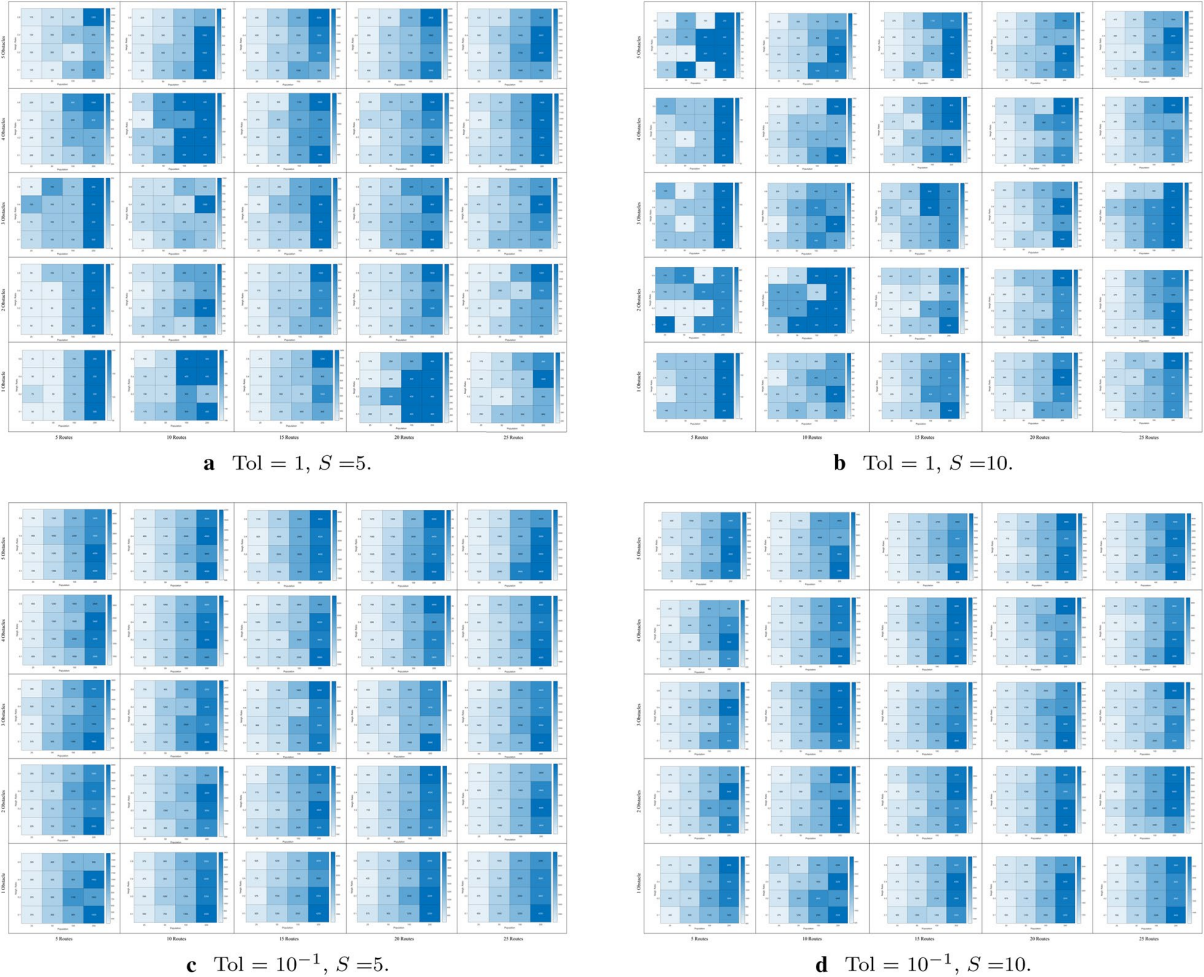
Number of evaluations required to achieve convergence in route bundling, in which darker colors imply large number of evaluations. The x-axis of each heatmap denotes the population size |**P**|, whereas the y-axis of each heatmap denotes the neighborhood scaling factor $\eta$. Heatmaps are arranged in a 2-dimensional grid, in which the horizontal axis of the grid denotes the number of edges |*E*| in the bipartite network and the vertical axis of the grid denotes the number of obstacles in the map. In this arrangement, heatmaps located at bottom/left (top/right) of the grid imply simple (complex) route bundling scenarios

**Fig. 11:**
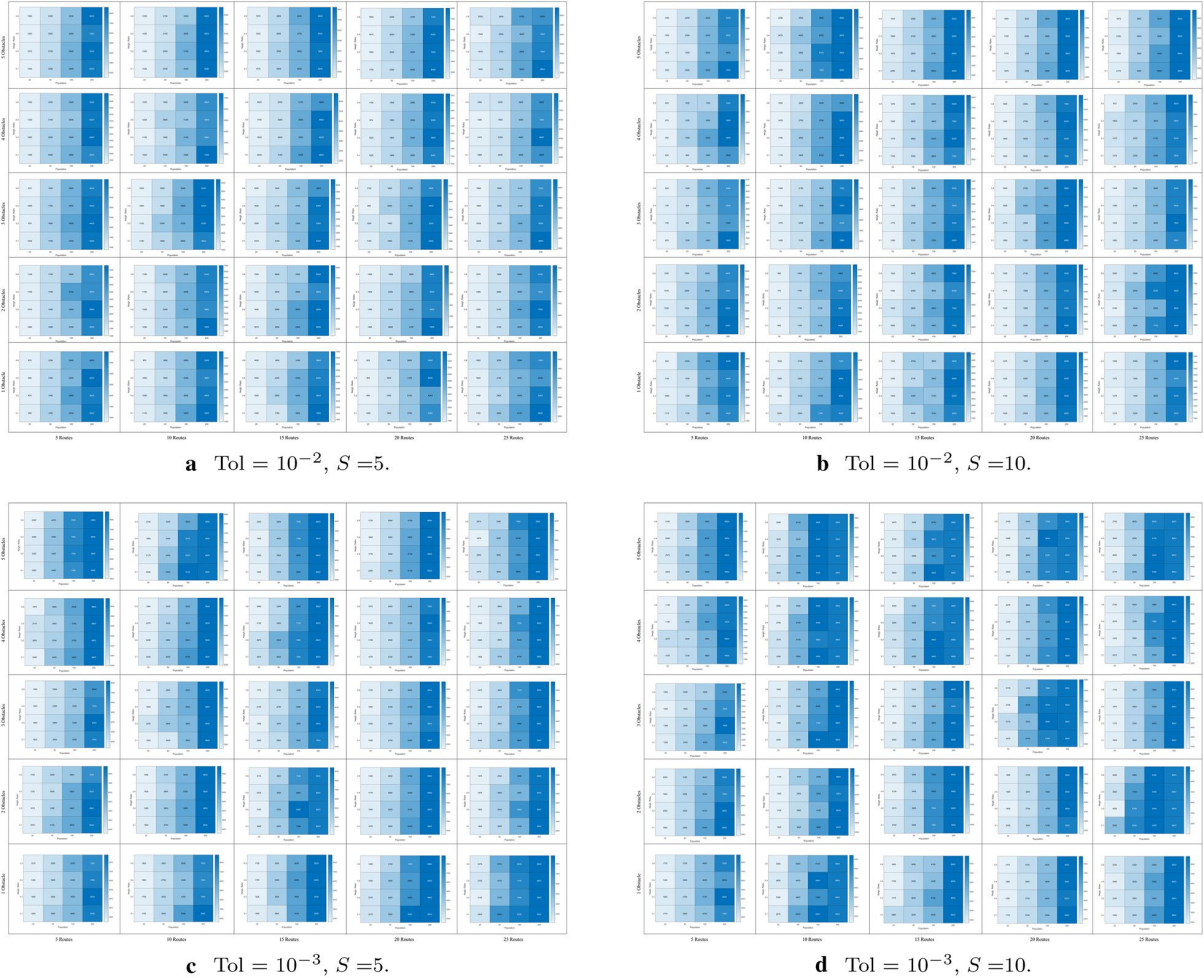
Number of evaluations required to achieve convergence in route bundling, in which darker colors imply large number of evaluations. The x-axis of each heatmap denotes the population size |**P**|, whereas the y-axis of each heatmap denotes the neighborhood scaling factor $\eta$. Heatmaps are arranged in a 2-dimensional grid, in which the horizontal axis of the grid denotes the number of edges |*E*| in the bipartite network, and the vertical axis of the grid denotes the number of obstacles in the map. In this arrangement, heatmaps located at bottom/left (top/right) of the grid imply simple (complex) route bundling scenarios

### Convergence

In order to show the kind of tree structures obtained in the route bundling process, Figs. 6 and 7 show the obtained route bundles in polygonal domains with obstacles of 5 and 10 sides, respectively. In order to show the efficiency of the proposed method, Figs. 8 and 9 show the convergence characteristics. Note that these figures are arranged in a grid in which the horizontal axis shows the number of edges |*E*| in the bipartite graph, and the vertical axis shows the number of obstacles in the polygonal map. In these figures, for the sake of simplicity, the values of ${\mathbf P} = 25$ and $\eta = 0.2$ are used; other values as denoted in Table 2 are discussed in subsequent sections.

In regard to the obtained route bundles, we can confirm the following facts:

- Regardless of the configuration of the polygonal map and the structure of the bipartite graph, it is possible to generate tree structures representing the bundled routes which aim at minimizing the global distance metric.
- The location of the anchoring points of the bundled routes is close to, but not necessarily at, the center of the origin and destination pairs of the bipartite graph.
- The route between the anchoring points of the bundled routes is not necessarily a straight line, and, regardless of increasing the number of edges in the bipartite graph, the routes between the anchoring points and the topologies of route bundles are structurally similar, but not equivalent. This is due to the fact of having edges with close origin–destination pairs.

The above observations has important implications to extend our proposed method in the following ways:

- instead of using arbitrary initial solutions in the optimization algorithm, it may be possible to compute the initial solutions of *x* which are close to the center/centroid of the origin and destination pairs, and
- it may be possible to use pre-computed routes between the anchoring points as initial solutions whenever the number of edges is expected to increase, since these routes are expected to be structurally similar.

The above are foundational insights to enable even faster convergence to the optimal solutions.

Furthermore, in regard to the convergence behavior of the Differential Evolution algorithm, Figs. 8 and  9 show the convergence behavior of the optimization algorithm over 15 independent runs. By observing these figures, it is possible to confirm the following facts:

- Regardless of the configuration of the polygonal maps and the structure of bipartite graphs, it is possible to converge to the bundled routes minimizing a global distance metric within 1000 function evaluations and 15 independent runs.
- Increasing the number of edges has a natural effect on increasing the distance metric by some small factor smaller than 1. This observation is in line with our above insights on the structural similarity of route bundles when increasing the number of edges.
- The convergence behavior of each simulation is different due to the heuristic nature of solution sampling in Differential Evolution, and the independent arbitrary initialization at each independent run. Note that it is imperative to use different arbitrary initializations in order to evaluate our approach exhaustively under diverse initialization conditions.

The above results imply the feasibility and efficiency to obtain optimal route bundles in polygonal maps with both convex and non-convex obstacles.

It is important to note that since obtaining a mathematical proof of convergence is unfeasible due to the heuristic nature of Differential Evolution, we argue that our converged results are approximations to the true global optima. Yet, our study is able to provide insights when Differential Evolution is used to tackle the route bundling problem under complex environments and diverse initialization conditions. Studying the theoretical convergence under non-heuristic algorithmic schemes is in our future agenda.

**Fig. 12:**
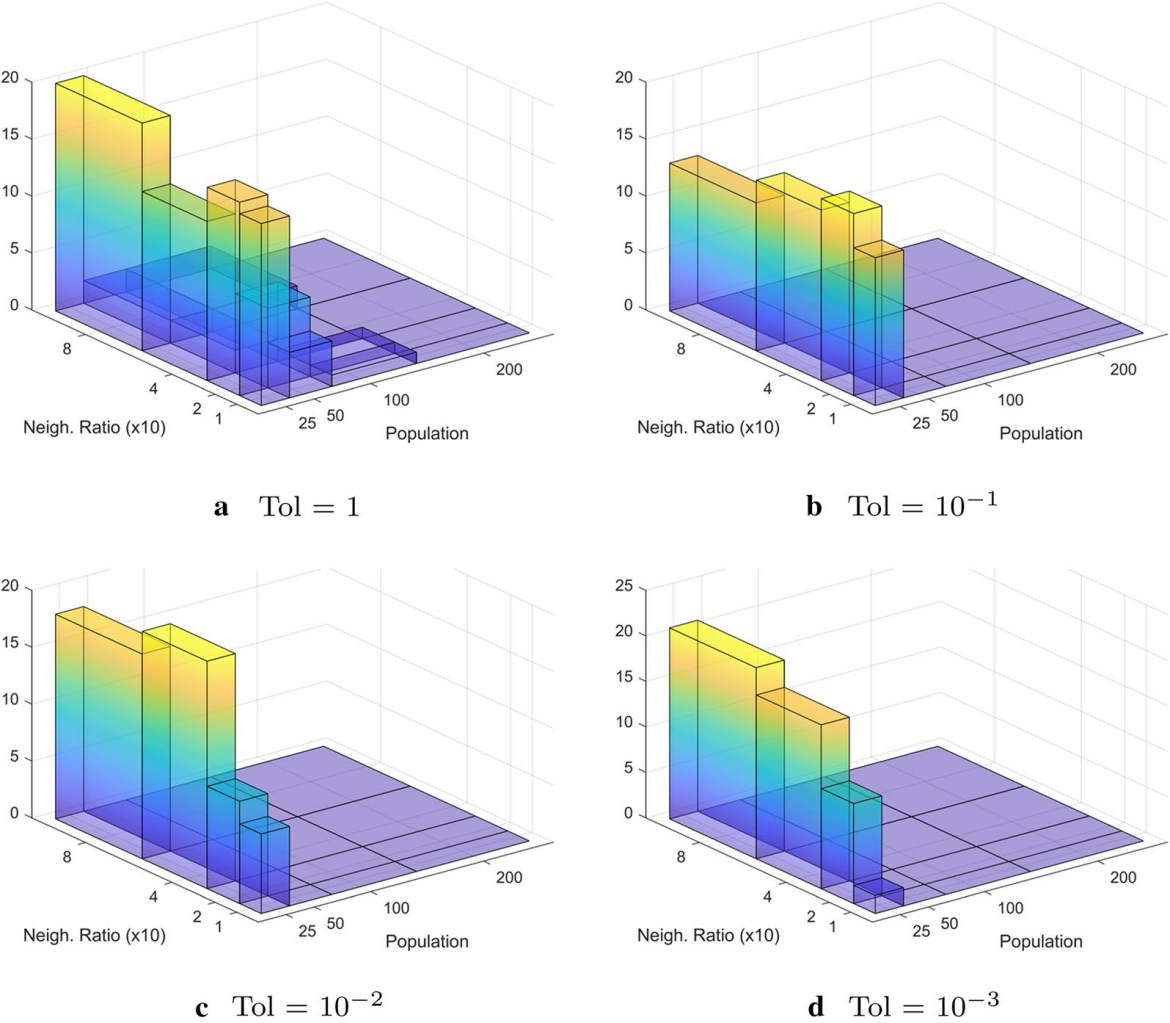
Histogram of $(|{\mathbf P} |, \eta )$ representing the *best* population size $|{\mathbf P} |$ and the *best* neighborhood scaling factor $\eta$ for all scenarios. Here, the meaning of *best* implies the tuple $(|{\mathbf P} |, \eta )$ which achieves the smallest number of evaluations to converge given a tolerance value. **a** The case when convergence is evaluated using $\hbox {Tol }= 1$. **b** The case when convergence is evaluated using $\hbox {Tol }= 10^{-1}$. **c** The case when convergence is evaluated using $\hbox {Tol }= 10^{-2}$. **d** The case when convergence is evaluated using $\hbox {Tol }= 10^{-3}$

Furthermore, in order to show the convergence at finer scale under different number of obstacles, routes, complexity of map, population size and neighborhood ratio, Figs. 10 and  11 show the required number of evaluations to achieve convergence. In these figures, the achievement of convergence is computed from the comparison of (1) the average difference of the cost function within 5 units of the convergence time series, to (2) the tolerance for convergence, which is a user-defined value rather than an optimization variable since it depends on the granularity of the polygonal map (maps requiring higher granularity imply finer and lower values of convergence tolerance).

In Figs. 10 and 11, for each number of routes and number of obstacles in the map, the heatmaps represent the number of evaluations required to achieve convergence for route bundling. Here, for each heatmap, **darker** colors imply large number of evaluations (max. number of evaluations is provided in the right side of each heatmap). Also, the x-axis of each heatmap represents the population size $|{\mathbf P} | = \{25, 50, 100, 200\}$ in Differential Evolution, whereas the y-axis of each heatmap denotes the neighborhood scaling factor $\eta \in \{0.1, 0.2, 0.4, 0.8\}$. Heatmaps are arranged in a 2-dimensional grid, in which the horizontal axis of the grid denotes the number of edges $|E| \in \{5, 10, 15, 20, 25\}$ in the input bipartite network, and the vertical axis of the grid denotes the number of obstacles in the map $\in \{1, 2, 3, 4, 5\}$. By this arrangement, heatmaps located at bottom/left grid imply simple route bundling scenarios, while heatmaps located at the top/right imply complex scenarios.

Then, by looking the results of Figs. 10 and  11, we observe that our proposed approach achieves faster convergence when using smaller populations ($|{\mathbf P} | = 25$) in most of the cases. We believe this is due to the fact of using a convex representation and a ring topology in Differential Evolution: whereas the convex representation helps sampling and evaluating unique solutions in the search space, the ring topology helps exploring the search space in areas close to the sampled solution. Thus, large populations or large neighborhood size has a detriment effect in widening the sampling space, which implies increasing the computational budget, and thus the required number of function evaluations to achieve convergence.

### Population and neighborhood size

Based on the above observations, the reader may wonder: what are good values of population size $|{\mathbf P} |$ and neighborhood scaling factor $\eta$? In order to answer this question, Fig. 12 shows the histogram of ($|{\mathbf P} |$, $\eta$) for the fastest converged values. In this figure, the x-axis of the histogram denotes the population size $|{\mathbf P} |$ and the y-axis of the histogram denotes the neighborhood ratio $\eta$. The histograms are based on the number of times in which the tuple ($|{\mathbf P} |$, $\eta$) achieved the smallest number of evaluations to achieve convergence. By observing Fig. 12, and in line of the above observations, for any value of evaluated tolerance for convergence in $\{1, 10^{-1}, 10^{-2}, 10^{-3}\}$ , *smaller populations are always beneficial*. In regard to the neighborhood size, for convergence tolerances being $10^{-1}$ or $10^{-2}$, the neighborhood scaling factor $\eta = 0.2$ is always beneficial, whereas for convergence tolerances being $10^{-3}$, the neighborhood scaling factor $\eta = 0.8$ is beneficial. These observations occur due to the fact of smaller tolerances implying the need to explore the search space at finer scale, thus higher neighborhood scaling factor $\eta$ enables the effective sampling of the search space without the need to increase population size (which would induce in unwanted space memory overhead). These results show that our proposed approach performs the heuristic search efficiently by using small populations.

### Algorithmic variants

The use of neighborhood and convex representation in Differential Evolution are relevant components in our proposed approach. Thus, in order to study the performance of these components in our proposed heuristic algorithm, we compared the following four variants:

- DENC: Differential Evolution with Neighborhood and Convex Encoding. In this scheme, we use a neighborhood with $\eta = 0.2$, based on our above observations, and the Convex Encoding denoted by the 6-dimensional tuple in Eq. 5 (described by “Representation of bundled routes” section).
- DEN: Differential Evolution with Neighborhood Only. In this scheme, we use a neighborhood with $\eta = 0.2$, based on our above observations, and the encoding denoted by the 4-dimensional tuple in Eq. 2, which is a simple representation yet computationally more expensive due to the fact of requiring checks of point inside polygon per every sampled solution to ensure *feasible* route bundles (coordinates are to be outside of obstacles). In this scenario, the cost function is computed as follows:
  17 $$\begin{aligned} F(x)= & {} {\left\{ \begin{array}{ll} G(x) &{}\, P, Q \in {\mathrm{free\,space}}\\ \infty &{} {\mathrm{otherwise}} \end{array}\right. } \end{aligned}$$
  18 $$\begin{aligned} G(x)= & {} \sum _{e \in E}d(e_o,P) + d(P,Q) + \sum _{e \in E}d(Q,e_d) \end{aligned}$$
  where *d*(*a*, *b*) is the Euclidean obstacle-free shortest distance metric between points *a* and *b*, $e_o$ is the *Cartesian *coordinate of the *origin* node of the edge $e \in E$, $e_d$ is the *Cartesian* coordinate of the *destination* node of the edge $e \in E$, and *P* and *Q* are the *Cartesian* coordinates of anchoring points being closer to the *origin* $e_o$ and *destination* $e_d$, respectively. The condition $P, Q \in$ *free space* satisfies that both *P* and *Q* are outside of the polygonal obstacles. Note that in this scheme, the Delaunay triangulation is not a requirement since all points are in $\mathbb {R}^2$.
- DEC, Differential Evolution with Convex Encoding Only. In this scheme, we use the Convex Encoding denoted by the 6-dimensional tuple in Eq. 5 (described by “Representation of bundled routes” section); yet, we avoid using neighborhood concepts, and thus the mutant vector $v_t$ (denoted by Eq. 13) is computed without the local interpolation vector, as follows:
  19 $$\begin{aligned} v_t = w^{x_t}.g_t \end{aligned}$$
  In the above, we keep the weight to be multiplying the global interpolation vector in order to enable individual self-adaptation.
- DE: Differential Evolution without Neighborhood nor Convex Encoding. In this scheme, we avoid using neighborhood concepts as well as the Convex Encoding. Therefore, the mutant vector $v_t$ is computed by Eq. 19, the encoding is depicted by the 4-dimensional tuple in Eq. 2, and the cost function is computed by Eq. 18.

**Fig. 13:**
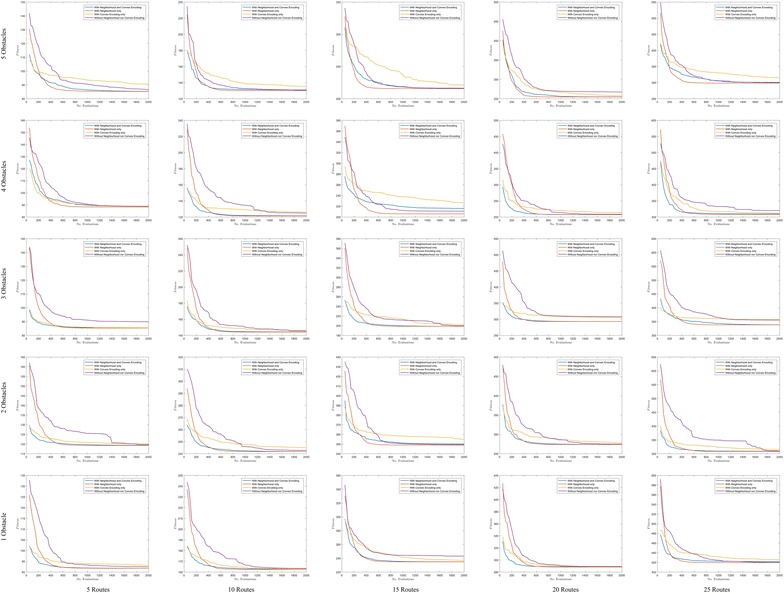
Comparison of convergence in polygonal domains of **S = 5 sides**

**Fig. 14:**
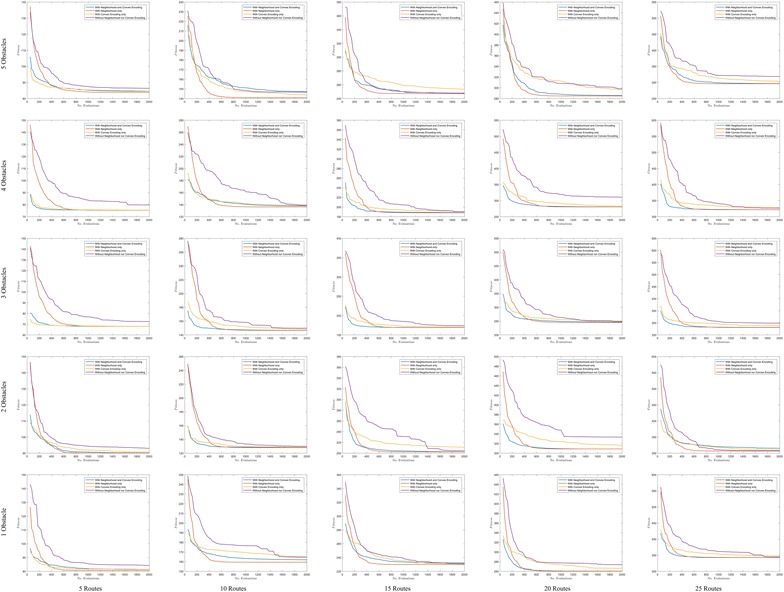
Comparison of convergence in polygonal domains of **S = 10 sides**

In the above described variants, we used the population size as $|{\mathbf P} | = 25$, based on our above observations describing the superiority of smaller populations. All other parameters in Differential Evolution are kept constant as described by Table 2.

In order to evaluate and compare the performance of the above described variants, Figs. 13 and 14 show the comparison of the average convergence behavior over 15 independent runs up to 2000 evaluations. The convergence figures are arranged in a 2-dimensional grid in which the x-axis portrays the number of edges in the input bipartite graph, and the y-axis portrays the number obstacles in the map. Thus, by looking at Figs. 13 and  14 we can observe the following facts:

- In all cases, DENC and DEC have better solutions during the initialization phase (when the number of evaluations is up to $|{\mathbf P} | = 25$) and the first number of evaluations (up to 200 in all cases). This occurs due to that fact of DENC and DEC using the convex representation which ensures sampling feasible points, always. Conversely, DE and DEN require additional number of evaluations (and checks of point inside polygon) in order to sample and evaluate feasible solutions.
- In all cases, either DENC or DEN has better convergence performance compared to DE. And in 26 out of 50 cases, DE has issues in stagnation. These observations imply that Differential Evolution using the interpolation vectors in the global and local neighborhood alone, or embedded with the convex representation, is useful not only to allow faster convergence, but also to allow escaping from stagnation. This occurs due to the fact of generating feasible solutions (by the convex representation), and due to the fact of self-balancing between directions toward the best in the population, and directions toward the best in the local neighborhood.
- Furthermore, in 10 out of 50 cases, DEC has issues of stagnation, which is in line with the above insights. Since DEC uses no information of the local neighborhood, sampled solutions will stagnate in directions close to the global best. Then, without any explorative factor, DEC is likely to stagnate. Thus, as the above observations indicate, the use of neighborhood enables to add an explorative factor to avoid stagnation.

Further computational experiments using large number of edges and diverse obstacle configurations reminiscent of outdoor environments are in our agenda. Also, although the main focus of this paper is environments in 2D, the extension of 3D is straightforward since:

- Delaunay triangulation in 3D takes in the worst case $O(n^2)$, and in the expected case can be even *O*(*n*).
- Differential Evolution can sample in an 8-dimensional tuple (for a convex representation) or 6-dimensional tuple (for a non-convex representation).
- Path planning in 3D is possible by either geometric or point cloud approaches.

Also, we aim at dealing with dynamic environments in our future work. A simple extension would consider the following principles:

- Obstacle geometry have minor changes over small time intervals, thus, to obtain the next optimal bundle, instead of using arbitrary initialization over the entire search space, it is possible to initialize candidate solutions with small perturbations to the current converged solutions and run Differential Evolution over a small number of evaluations. In this way, it becomes possible to quickly update the topology of the route bundles when changes in obstacle geometry are expected occur.
- The same strategy can be used whenever the locations of origin and destination in the bipartite graph are expected to occur.
- In order to realize a fast response time, it is possible to use multi-core computing since Differential Evolution supports parallelization inherently.

Also, in our agenda remains the use of undirected and directed graphs [27, 28], modularity by combinatorial groupings of nodes and edges [29, 30] to build trees with increased depth (to improve scalability) and its application to network design [31, 32]. Furthermore, it remains in our agenda the study of changing structures during network optimization, and the use of concurrent exploration and exploitation. Last but no least, the extension to generate curved and collision-free navigation bundles in cluttered environments [33–35] is left for future work. Our results offer building blocks to further advance toward developing global network optimization with convex, flexible and scalable representations.

## Conclusions

In this paper, we have proposed a method for searching optimal route bundles based on a self-adaptive class of Differential Evolution and a convex representation. The basic idea of our approach is to sample over a triangulated search space by using self-adaptive interpolation vectors. And the unique point of our proposed method is the possibility to balance exploration and exploitation while sampling arbitrary points in a convex search space of route bundles. Then, it becomes possible the rendering of feasible route bundles efficiently since: (1) absence of overlaps with obstacles is guaranteed, and (2) computation of point inside polygon is explicitly avoided. Computational experiments involving more than 12,000 route bundling cases and 11,250,000,000 evaluations of path planning in a diverse class of polygonal domains show that (1) it is possible to obtain bundled routes with an optimized global distance metric via a reasonable number of sample evaluations, (2) the convergence towards the optimal solutions is possible over independent runs, (3) smaller populations are always beneficial, and (4) the interpolation vectors in the global and local neighborhood and the convex representation are useful not only to allow faster convergence, but also to allow escaping from stagnation.

In our future work, we aim at using polygonal environments reminiscent of outdoor configurations in which the number of edges of the bipartite network is allowed to increase. Also, in our future endeavors, we aim at exploring the generalization ability in dynamic environments, where both the input bipartite graph and the polygonal obstacles are allowed to change. We believe our approach opens new frontiers to further develop compounded and global path-planning algorithms via gradient-free optimization algorithms and convex representations of the search space.

## Footnotes

1
$$5\times 5\times 2\times 4\times 4\times 15.$$

2 considering population size, number of independent runs, number of max. evaluations for all experimental conditions.
